# Severe Human Intestinal Spirochetosis: An Unusual Cause of Diffuse Colonic Ulcerations in a Patient Living with HIV

**DOI:** 10.1155/2019/1504079

**Published:** 2019-10-15

**Authors:** T. A. Ajose, J. Aniekwena, V. S. Effoe, M. Simien

**Affiliations:** ^1^Division of General Internal Medicine, Department of Medicine, Morehouse School of Medicine, Atlanta, GA, USA; ^2^Division of Gastroenterology and Hepatology, Department of Medicine, Morehouse School of Medicine, Atlanta, GA, USA

## Abstract

We describe a case of a homosexual male with human immunodeficiency virus (HIV) and CD4 count of 246 presenting with acute severe bloody diarrhea. Infectious work up was negative, and colonoscopy revealed severe diffuse colonic ulcerations. Histopathologic analysis and Treponemal pallidum immunostaining confirmed the diagnosis of intestinal spirochetosis. There was no evidence of co-infection with other pathogens. His symptoms completely resolved after a 14-day course of metronidazole. This case is notable as colonic ulceration of any severity in patients living with HIV is rarely identified with intestinal spirochetosis. Hence, it should be considered in the differential diagnosis of colonic ulcerations.

## 1. Introduction

Colonic ulceration in HIV infected individuals is commonly attributed to infectious colitis (viral, bacterial, and fungal), HIV and/or inflammatory bowel disease. Among bacterial causes, pathogens like salmonella, shigella, campylobacter, Yersinia and Neisseria gonorrhea predominate. With the advent of antiretroviral therapy (ART), the incidence of infectious causes in those with low CD4 counts have significantly declined [1]. Intestinal spirochetosis is however gaining clinical significance as an infectious cause of colonic ulceration in this population. However, data on cases with colonic ulcerations remains sparse. Herein, we present a unique case of a HIV-infected male with diffuse colonic ulcerations attributed solely to intestinal spirochetosis.

## 2. Case Presentation

A 30-year-old African-American male with a medical history significant for HIV infection diagnosed 5 years prior (not on ART) presented to the Emergency department with a 2-week history of severe watery diarrhea. On average, he had 4 stools daily, which were initially nonbloody, but became bloody one-day prior to his presentation with an increased frequency of 10–12 stools. Diarrhea was associated with severe, crampy left lower abdominal pain, chills and drenching sweats. Personal history was notable for recent receptive anal intercourse. Colonoscopy done 2 years prior for similar symptoms, was normal.

On physical exam, his vital signs were: BP 111/88 mmHg, HR 101 bpm, RR 14 breaths/min, Temp 37.4°C, and oxygen saturation 97% on room air. He had dry oral mucous membranes and tenderness was elicited in the left lower abdomen. No pallor was noted. The rest of his exam was unremarkable. Laboratory testing revealed erythrocyte sedimentation rate 41 mm/hr, C-reactive protein 4.9 mg/dl and a CD4 count of 246. No leukocytosis or anemia. Serum amylase, serum lipase, lactic acid, liver and renal function testing were all unremarkable. Infectious work-up for his diarrhea, including stool ova, and parasite, Clostridium difficile toxin stool assay, stool culture, rapid plasma reagin, Cryptosporidium antigen, rotavirus antigen and Giardia antigen were all negative. A contrast-enhanced computed tomographic scan of his abdomen was unrevealing for any bowel pathology. He was admitted to the general floors for volume resuscitation and pain control.

**Figure 1:**
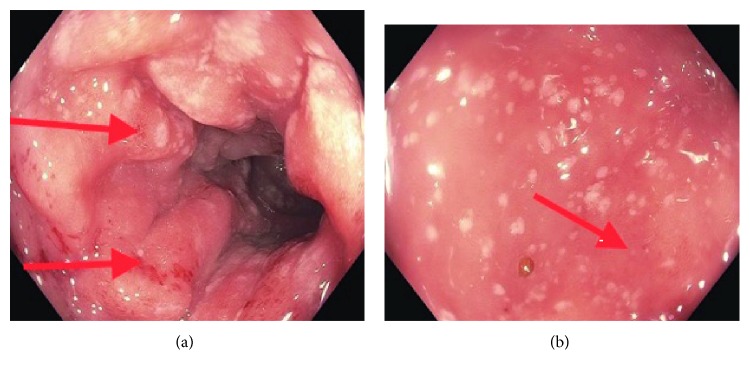
Colonoscopy showing condylomatous changes in the rectum and severe diffuse punctate ulcerations and erythema involving the entire colon.

**Figure 2:**
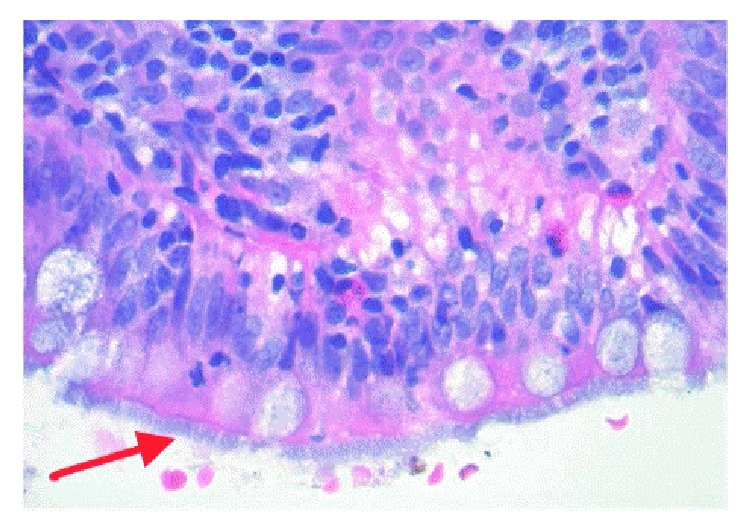
Hematoxylin and Eosin stain. Light microscopy of colonic biopsy specimens revealed intestinal spirochetes with nonspecific colitis in the entire colon and rectum.

**Figure 3:**
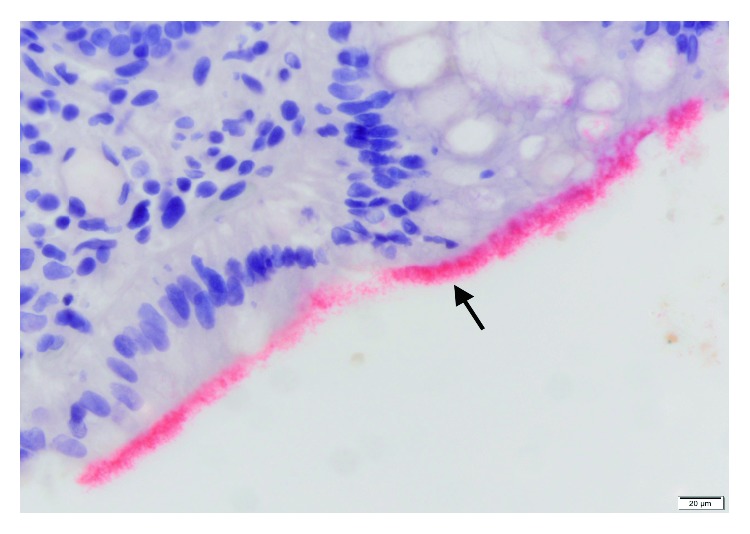
Treponemal Pallidum Immunostain. Spirochetes (red band) forming a false brush border over surface epithelium.

Given severity of bloody diarrhea, a colonoscopy was performed which revealed condylomatous changes in the rectum and severe diffuse punctate ulcerations and erythema involving the entire colon but worse in the left colon (Figures 1(a) and 1(b)). Light microscopy with Hematoxylin and Eosin staining of colonic biopsy specimens revealed intestinal spirochetes with non-specific colitis in the entire colon and rectum (Figure 2). Silver staining revealed similar findings as well (see Supplementary [Supplementary-material supplementary-material-1]). Treponemal pallidum immunostaining showed spirochetes forming a false brush border appearance over surface epithelium (Figure 3). No other pathogen was identified. A diagnosis of noninvasive intestinal spirochetosis was made. He received a 14-day course of oral Metronidazole and he witnessed complete resolution of his symptoms. ART was commenced on discharge. Repeat colonoscopy was not pursued due to symptom resolution and patient's preference.

## 3. Discussion

Human intestinal spirochetes consist of a heterogenous group of gram-negative bacteria which include *Brachyspira pilosicoli* and *Brachyspira aalborgi*. The prevalence of intestinal spirochetosis is high in developing countries, but in the developed world, rates are highest amongst homosexual individuals with or without HIV [2] raising the question of possible sexual transmission. *B. pilosicoli* predominates in HIV-infected persons and is considered an opportunistic pathogen [3]. Though the pathogenesis of this disease remains poorly understood, a study has demonstrated that strains of *B. pilosicoli* vary in their ability to attach to Caco-2 cells and the attached Caco-2 cells undergo a series of changes, including accumulation of actin at the cell junctions, disruption to the cell membrane, apoptosis, and up-regulation of IL-1b and IL-8 [4]. *B. pilosicoli* surface lipoproteins may be involved in facilitating this attachment by undergoing protein-protein interactions with specific receptors on the cell surface. The appearance of a “false brush border” is shown if a sufficient number of cells become attached and where massive colonization occurs, it may cause a physical impedance to water and electrolyte absorption through the colonic enterocytes and hence contribute to diarrhea [5].

Most cases are asymptomatic, but our patient presented with symptoms of abdominal pain, diarrhea and rectal bleeding, which has been described in other patients [6]. Intestinal spirochetosis is mostly detected as an incidental finding on screening colonoscopy, making its role as a pathogen unclear. Histologically, they assume a band-like growth over the luminal surface of the colon, giving a brush border appearance. This histologic appearance depicts the noninvasive form as seen in our patient. Histologic techniques applied include hematoxylin and eosin (H&E) stain, silver stains (e.g., Warthy-Starry) and immunostains [7].

Colonoscopy remains an invaluable aid to diagnosis. Mucosal appearances are highly variable; from normal appearing (most common) to erythematous, polypoid and mucosal erosions [8, 9]. Colonic ulcerations are less described in intestinal spirochetosis and are more commonly seen in other forms of bacterial colitis (salmonella, shigella, campylobacter, Yersinia, and Neisseria gonorrhea) [10], viral colitis (CMV, HSV, and HIV enteropathy), fungal colitis (cryptococcosis and histoplasmosis) and inflammatory bowel disease. Co-infection with these enteric pathogens which cause colonic ulceration is common [11, 12] hence prompting the need to exclude alternative causes. Despite extensive evaluation, no other etiology could account for the findings seen in our patient.

In a case report by Kostman et al. [13], diffuse colonic ulceration was described in one patient with Acquired immunodeficiency syndrome (AIDS). This patient was found to have invasive intestinal spirochetosis as confirmed by histologic studies. In comparison to our case, our patient was not severely immunosuppressed, and he had the noninvasive form of spirochetosis. Hence, it is uncertain the role invasion plays with colonic ulcer development.

Factors that lead to mucosal damage after colonization is unclear. Cooper et al. [14] put forward various mechanisms which include higher degree of mucosal attachment, recurrence of infection at same sites, organism burden and direct effect of trauma from sexual practices. Law et al. [15] also suggest a direct immunosuppressive effect on the mucosal immune-reactive cells by HIV. As far as we know, this was the first episode of intestinal spirochetosis in our patient and prior colonoscopy was normal.

Frequent association with other intestinal diseases, including carcinoma, colonic polyps, and ulcerative colitis have been reported [16]. The aforementioned conditions could lead to immunosuppression, thus creating a favorable environment for spirochetes to invade the colonic epithelium. Our patient lacked these findings and his immunosuppressive state was induced by HIV.

The drug of choice is metronidazole and our patient witnessed a satisfactory response to this first line therapy.

## 4. Conclusion

Intestinal spirochetosis should be considered in all cases of colonic ulcerations in HIV patients irrespective of the degree of immunosuppression as effective treatment is available. It is recommended that alternate causes of colonic ulceration be excluded as co-infection with other enteric pathogens is common.

## Supplementary Materials

Supplementary Materials Supplementary Figure S1: silver stain showing spirochetes (red arrow) over apical epithelial surface with nonspecific colitis.
