# Supplementary figures and images for: Severe Human Intestinal Spirochetosis: An Unusual Cause of Diffuse Colonic Ulcerations in a Patient Living with HIV
Source: Case Rep Gastrointest Med. 2019 Oct 15;2019:1504079. doi: 10.1155/2019/1504079 (PMC6815633; doi:10.1155/2019/1504079)

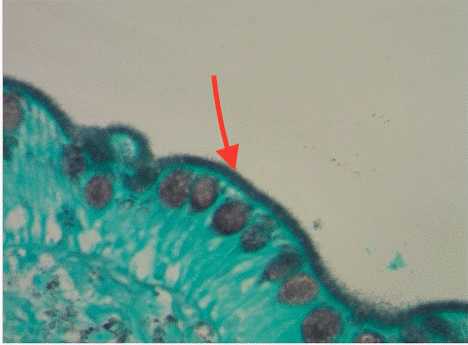

Supplement: Supplementary Materials — Supplementary Figure S1: silver stain showing spirochetes (red arrow) over apical epithelial surface with nonspecific colitis. [file 1504079.f1.gif]
